# Predicting ustekinumab treatment response in Crohn’s disease using pre-treatment biopsy images

**DOI:** 10.1093/bioinformatics/btaf301

**Published:** 2025-05-14

**Authors:** Chengfei Cai, Ruidong Chen, Jieyu Chen, Jun Li, Caiyun Lv, Yiping Jiao, Lanqing Wu, Juan Chen, Qi Sun, Qianyun Shi, Jun Xu, Wen Tang, Yao Liu

**Affiliations:** Jiangsu Key Laboratory of Intelligent Medical Image Computing, School of Artificial Intelligence, Nanjing University of Information Science and Technology, Nanjing 210044, China; School of Automation, Nanjing University of Information Science and Technology, Nanjing 210044, China; College of Information Engineering, Taizhou University, Taizhou 225300, China; Department of Gastroenterology, the Second Affiliated Hospital of Soochow University, Suzhou 215004, China; Department of Pathology, Nanjing Drum Tower Hospital, the Affiliated Hospital of Nanjing University Medical School, Nanjing 210008, China; Jiangsu Key Laboratory of Intelligent Medical Image Computing, School of Artificial Intelligence, Nanjing University of Information Science and Technology, Nanjing 210044, China; Department of Gastroenterology, the Second Affiliated Hospital of Soochow University, Suzhou 215004, China; Jiangsu Key Laboratory of Intelligent Medical Image Computing, School of Artificial Intelligence, Nanjing University of Information Science and Technology, Nanjing 210044, China; Department of Pathology and Pathophysiology, Medical College of Soochow University, Soochow University, Suzhou 215123, China; Department of Pathology and Pathophysiology, Medical College of Soochow University, Soochow University, Suzhou 215123, China; Department of Pathology, Nanjing Drum Tower Hospital, the Affiliated Hospital of Nanjing University Medical School, Nanjing 210008, China; Department of Pathology, Nanjing Drum Tower Hospital, the Affiliated Hospital of Nanjing University Medical School, Nanjing 210008, China; Jiangsu Key Laboratory of Intelligent Medical Image Computing, School of Artificial Intelligence, Nanjing University of Information Science and Technology, Nanjing 210044, China; Department of Gastroenterology, the Second Affiliated Hospital of Soochow University, Suzhou 215004, China; Department of Gastroenterology, the Second Affiliated Hospital of Soochow University, Suzhou 215004, China; Department of Pathology, Nanjing Drum Tower Hospital, the Affiliated Hospital of Nanjing University Medical School, Nanjing 210008, China; Department of Pathology and Pathophysiology, Medical College of Soochow University, Soochow University, Suzhou 215123, China

## Abstract

**Motivation:**

Crohn’s disease (CD) exhibits substantial variability in response to biological therapies such as ustekinumab (UST), a monoclonal antibody targeting interleukin-12/23. However, predicting individual treatment responses remains difficult due to the lack of reliable histopathological biomarkers and the morphological complexity of tissue. While recent deep learning methods have leveraged whole-slide images (WSIs), most lack effective mechanisms for selecting relevant regions and integrating patch-level evidence into robust patient-level predictions. Therefore, a framework that captures local histological cues and global tissue context is needed to improve prediction performance.

**Results:**

We propose a novel clustering-enhanced weakly supervised learning framework to predict UST treatment response from pre-treatment WSIs of CD patients. First, patches from WSIs were encoded using a pre-trained vision foundation model, and k-means clustering was applied to identify representative morphological patterns. Discriminative patches associated with treatment outcomes were selected via a DenseNet-based classifier, with Grad-CAM used to enhance interpretability. To aggregate patch-level predictions, we adopted a multi-instance learning approach, from which whole-slide features were extracted using both patch likelihood histograms and bag-of-words representations. These features were subsequently used to train a classifier for final response prediction. Experimental results on an independent test set demonstrated that our WSI-level model achieved superior predictive performance with an AUC of 0.938 (95% CI: 0.879–0.996), sensitivity of 0.951, and specificity of 0.825, outperforming baseline patch-level models. These findings suggest that our method enables accurate, interpretable, and scalable prediction of biological therapy response in CD, potentially supporting personalized treatment strategies in clinical settings.

**Availability and implementation:**

https://github.com/caicai2526/USTAIM.

## 1 Introduction

Crohn’s disease (CD) is a chronic inflammatory gastrointestinal disease that can affect any part of the digestive tract, from the mouth to the anus. It often involves the terminal ileum and colon (Baumgart and Sandborn 2012, Veauthier and Hornecker 2018, Ogino *et al.* 2020). It affects individuals of all age, with over 80% of patients diagnosed before the age of 40 (Baumgart and Sandborn 2012). Clinically, CD may lead to the development of strictures, fistulas, and abscesses. Within ten years of diagnosis, approximately 71% of patients require surgical resection of intestinal lesions (Bernell *et al.* 2000a). The recurrence rate of symptoms is estimated to be 40% within ten years after surgery (Bernell *et al.* 2000b), with an endoscopic recurrence rate of 85% within three years after surgery (Burr *et al.* 2019). Treatment of CD primarily involves nutritional support and pharmacotherapy, which includes corticosteroids, immunosuppressants, and biological agents. Anti-tumour necrosis factor agents, such as infliximab and adalimumab, effectively induce remission (Targan *et al.* 1997, Colombel *et al.* 2007). However, after a period of treatment, some patients experience disease relapse, necessitating a switch to alternative medications. Early identification of treatment response in CD patients is therefore crucial for disease monitoring and treatment strategy formulation.

Ustekinumab (UST) is a novel monoclonal antibody used to treat CD by inhibiting interleukin-12/23 (IL-12/23) signalling, thereby reducing inflammation (Feagan *et al.* 2016). It has shown efficacy in improving clinical symptoms, delaying surgery (Sandborn *et al.* 2012, Sandborn *et al.* 2018, Sands *et al.* 2019). However, its effectiveness varies among CD patients, necessitating close monitoring and potential treatment adjustment (Lamb and Duggan 2017). To predict UST response in CD patients, a gene expression prediction model was developed, achieving an AUC of 0.734 in the test set (He *et al.* 2021). In moderate to severe CD patients treated, COX proportional hazard analysis indicated a 66.7% probability of achieving clinical remission in moderate cases and 75% in severe cases (Park *et al.* 2023). These findings highlight the importance of drug monitoring in CD therapeutics, utilizing predictive modelling for assessing UST response and guiding clinical and endoscopic follow-ups for patients (Liefferinckx *et al.* 2023, Peyrin-Biroulet *et al.* 2024).

The wide application of AI technology in the biomedical field has had a huge impact on this field (Yu *et al.* 2018, Bi *et al.* 2019). In histopathology, the progress in oncology has been most pronounced (Van der Laak *et al.* 2021, Feng *et al.* 2022, Wang *et al.* 2023, Jiang *et al.* 2024). However, applying AI methods in clinical practice and clinical experiments still faces many challenges (LeCun *et al.* 2015). These challenges encompass the acquisition of sufficiently large and well-labelled training datasets, variations in image quality, and specimen collection methods necessitating extensive data normalization (Sola and Sevilla 1997, Clarke and Treanor 2017). Additionally, hardware constraints may arise when processing large, high-resolution images (Pinckaers *et al.* 2021). AI in gastroenterology is growing, with most using endoscopic data. Related studies using AI to predict treatment response in gastrointestinal tumours have shown that AI can capture subtle differences in tumour morphology (Kather *et al.* 2019a,b, Kumar *et al.* 2022, Zamanitajeddin *et al.* 2024). However, applying AI to non-neoplastic diseases like CD, characterized by diffuse lesions with morphology resembling normal tissue, presents unique challenges.

This study proposes a novel application of AI technology to assess treatment response in CD patients treated with UST based on histopathological images. The model predicts UST treatment outcomes using pathology images from pre-treatment endoscopic biopsies, analysed through serial biopsy sections. The model circumvents labelling challenges by employing feature domain clustering and multi-instance feature fusion modes, using patient-level UST response types as weakly supervised labels for training. Promising results on the test set highlight the potential of AI to augment clinical decisions in treating CD.

## 2 Materials and methods

### 2.1 Overview

**Figure 1.**
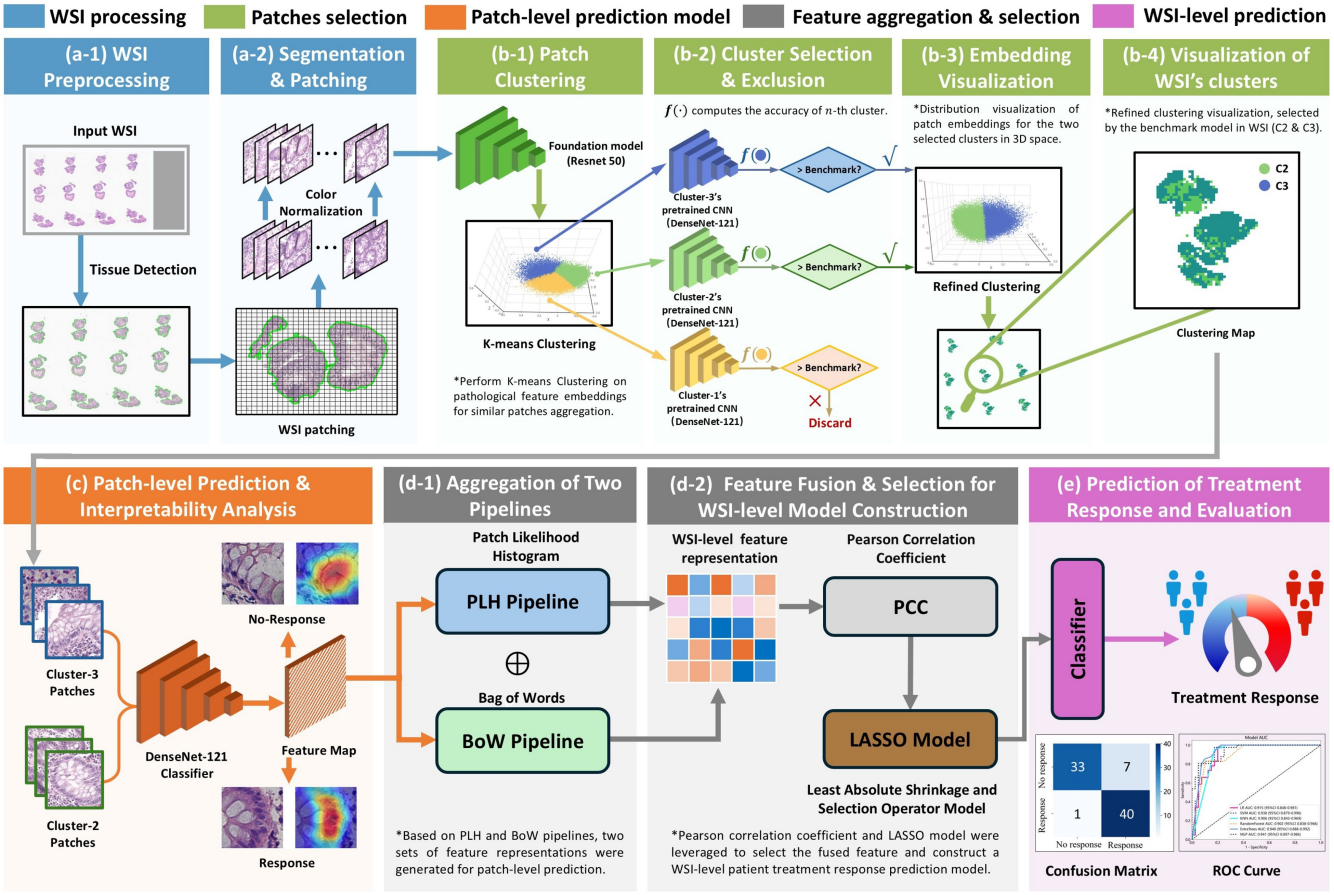
Overview of the clustering-enhanced weakly supervised learning framework for UST response in CD, which consists of five parts: WSI processing, patch selection, patch-level prediction, feature aggregation and selection, and WSI-level prediction. Figures (a-1) and (a-2) show tissue detection and stain normalization. Patch selection (Figures b-1 to b-4) involves feature extraction via a foundation model, k-means clustering, and identification of discriminative clusters outperforming a baseline. Figure (c) illustrates patch-level model construction. Figures (d-1) and (d-2) show PLH/BoW feature aggregation and selection. Figure (e) presents WSI-level prediction results, including the confusion matrix and ROC curve.

As shown in Fig. 1, the study’s prediction model framework consists of five main components: (i) preprocessing and standardization of WSI, (ii) construct a patch screening model to identify effective predictors of UST response, (iii) development of a patch-level UST response prediction model using selected patches, (iv) integration of multiple instances to aggregate predicted patch results and generate feature representations for each WSI, and (v) establishment of a WSI-level UST response prediction model. This approach forms the study’s core methodology, focusing on predicting treatment outcomes based on histopathological images.

### 2.2 Data collection and preparation

The experimental data for this study were obtained from the Second Affiliated Hospital of Soochow University, a total of 404 biopsy tissues from CD patients. Inclusion criteria for the study subjects: (i) all patients were diagnosed with CD; (ii) UST treatment was received; (iii) endoscopic examination at baseline indicated disease activity and the SES-CD score was more significant than 3 points; and (iv) follow-up time was at least >24 weeks, and endoscopic review was completed; refer to the ‘Chinese Consensus on Diagnosis and Treatment of Inflammatory Bowel Disease (2018 Beijing),’ combined with the patient’s clinical manifestations, endoscopy, imaging, pathological examination, and so on. Comprehensive diagnosis of CD. Exclusion criteria: (i) age under 18 years old; (ii) endoscopic review was incomplete; (iii) baseline data were incomplete; and (iv) other drugs were changed due to non-efficacy-related factors.

**Figure 2.**
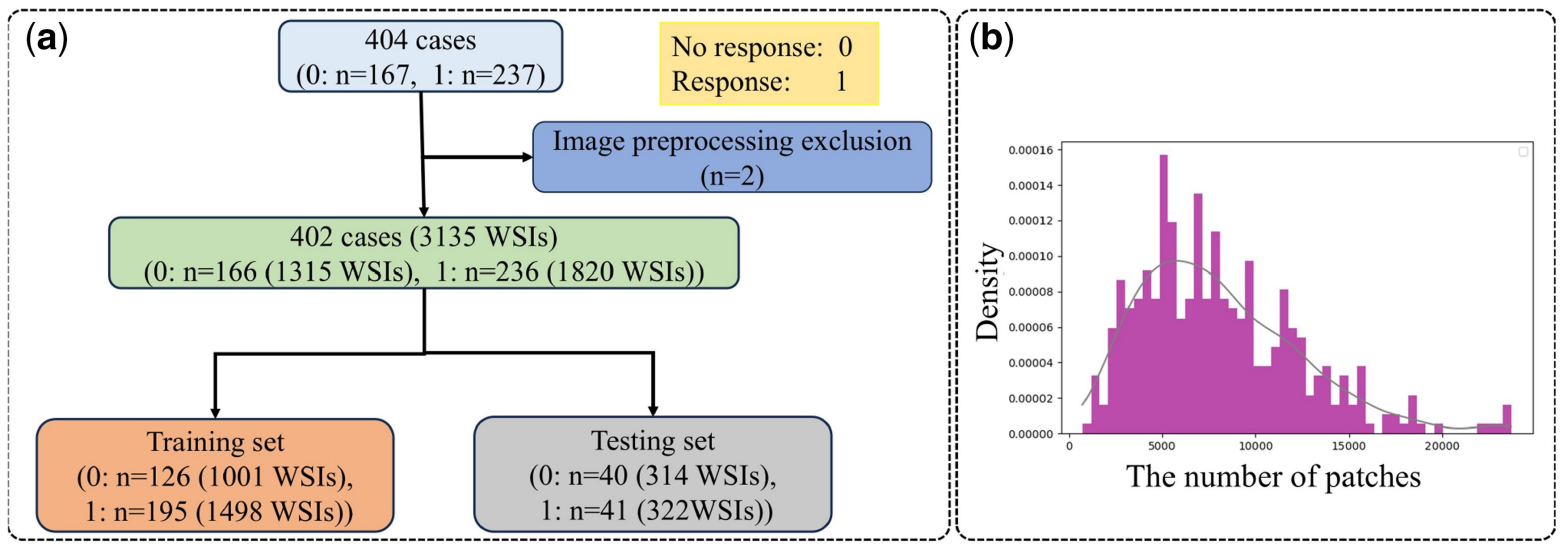
Dataset introduction. (a) Flowchart of dataset selection. A total of 404 biopsy samples were collected and digitized using image preprocessing methods by the scanner. Two biopsy samples were excluded, resulting in 402 samples used in this study. The dataset was randomly divided into a model set (*n* = 321) and a test set (*n *= 81). (b) Statistical analysis of the number of patches divided per sample.

During image preprocessing, two biopsy tissues did not meet experimental requirements because the biopsy tissues were too small, preventing effective image area extraction. The dataset used in the experimental process of this study included 402 biopsy tissues. All patients with biopsy samples were treated with UST, with 236 samples showing treatment response and 166 without treatment response. Each biopsy tissue sample had multiple serial sections, resulting in 1820 WSIs with treatment response and 1315 WSIs without treatment response. WSIs were digitized using a Leica GT450 scanner at 263 nm/pixel. For predicting drug response, the study randomly divided the data set into two groups (model set: test set = 8:2), with 321 biopsy tissue samples in the model set and 81 biopsy tissue samples in the independent test set. The data description is depicted in Fig. 2.

### 2.3 WSI preprocessing and normalization

Due to the large size of WSIs, often reaching the gigabyte level in storage, direct processing is challenging. This study utilizes a method similar to CLAM (Lu *et al.* 2021) for preprocessing. By taking out the image’s foreground, WSIs were preprocessed into 256×256 pixel patches at the maximum magnification of the WSI. At the same time, patches with more than 50% of the tissue area were selected. Consequently, the study generated a dataset of millions of patches, including more than 2.6 million in the model cohort and more than 590 000 in the test cohort. This study uses HE-stained pathological tissue images. To address issues like uneven staining and fading, the Vahadane *et al.* (2016) colour standardization method is employed for processing, as illustrated in Fig. 1a-1 and a-2.

### 2.4 Selection of patches for downstream analysis

#### 2.4.1 Patch clustering model for discriminative patch selection

This study clusters patches according to their phenotypes and identifies discriminative patches to select effective patches from those generated by WSIs. As shown in Fig. 1b-1, initially, 50 samples were randomly selected from the model cohort, comprising 29 response and 21 non-response samples. Considering that the original image may contain drug response phenotypes unrelated to their type, this study clusters patches in the feature domain. Through transfer learning, the Resnet50 model trained on the ImageNet dataset acts as a feature extractor, converting all patches into 2048-dimensional structured data through the average pooling layer. This study developed the K-means clustering algorithm on 329 045 patches from 50 biopsy tissue samples. The clustering quality was evaluated using the Calinski-Harabasz (CH) index. Patches in different clusters exhibited distinctive imaging patterns related to response, as depicted in Fig. 1b-1.

#### 2.4.2 Patch selection for patch-level prediction of UST response

**Figure 3.**
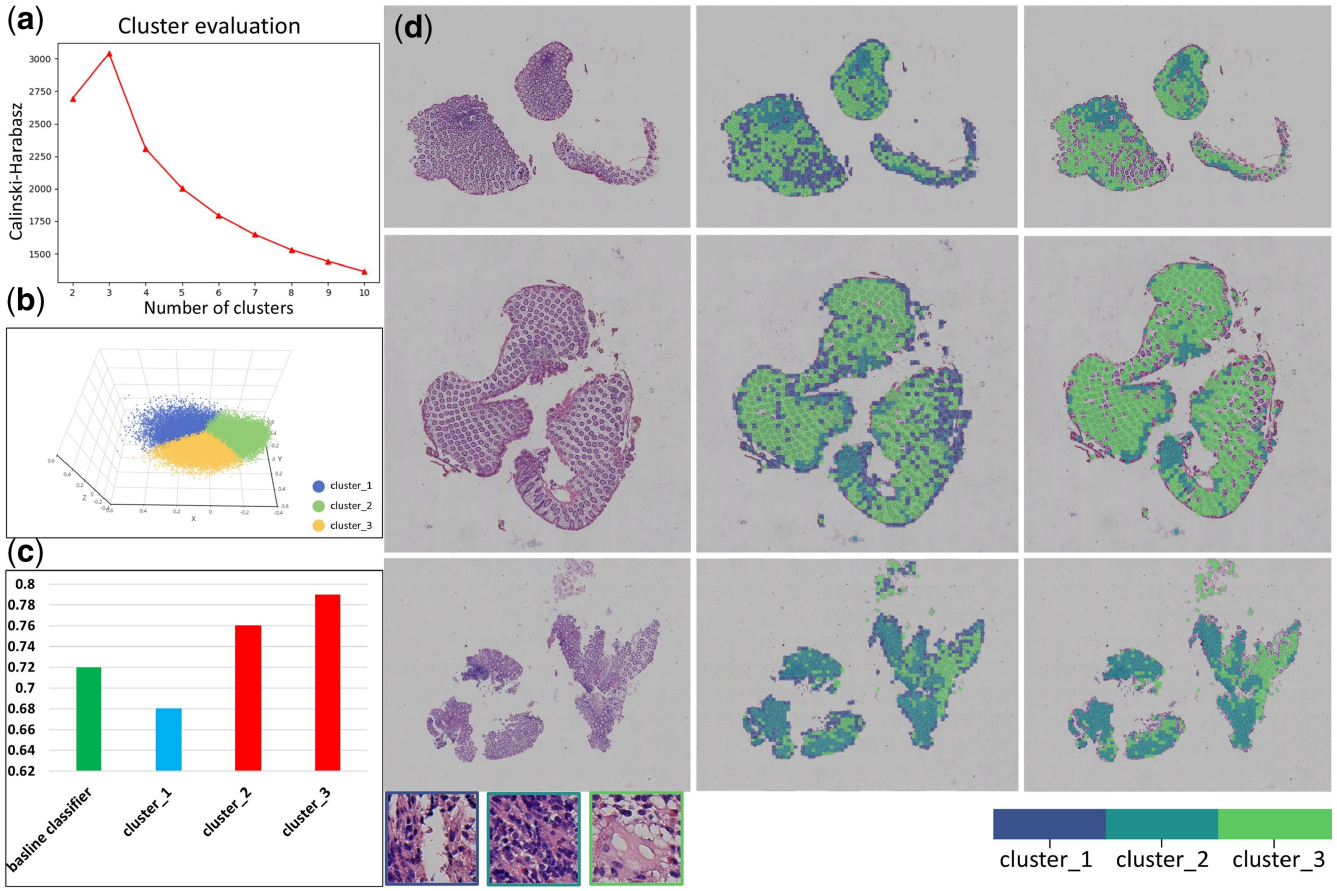
Results of patch clustering and selection. (a) CH index plot illustrating k-means clustering performance across 2–10. (b) Visualization depicting 3-category clustering of 50 random samples from the model cohort. (c) A histogram presenting patch-level classification accuracy for three independent clustering classifiers. Classifiers trained on clusters 2 and 3 demonstrate higher accuracy than the baseline classifier. Then, patches within clusters 2 and 3, corresponding to each patient, are selected for constructing a patient-level classifier. (d) Results of patch clustering and selection for three representative samples. Additional consecutive slices are shown in Supplementary Fig. S1. Images displayed from left to right: the original WSI, distribution of clustered patches, and the final selected patch in the cluster.

**Figure 4.**
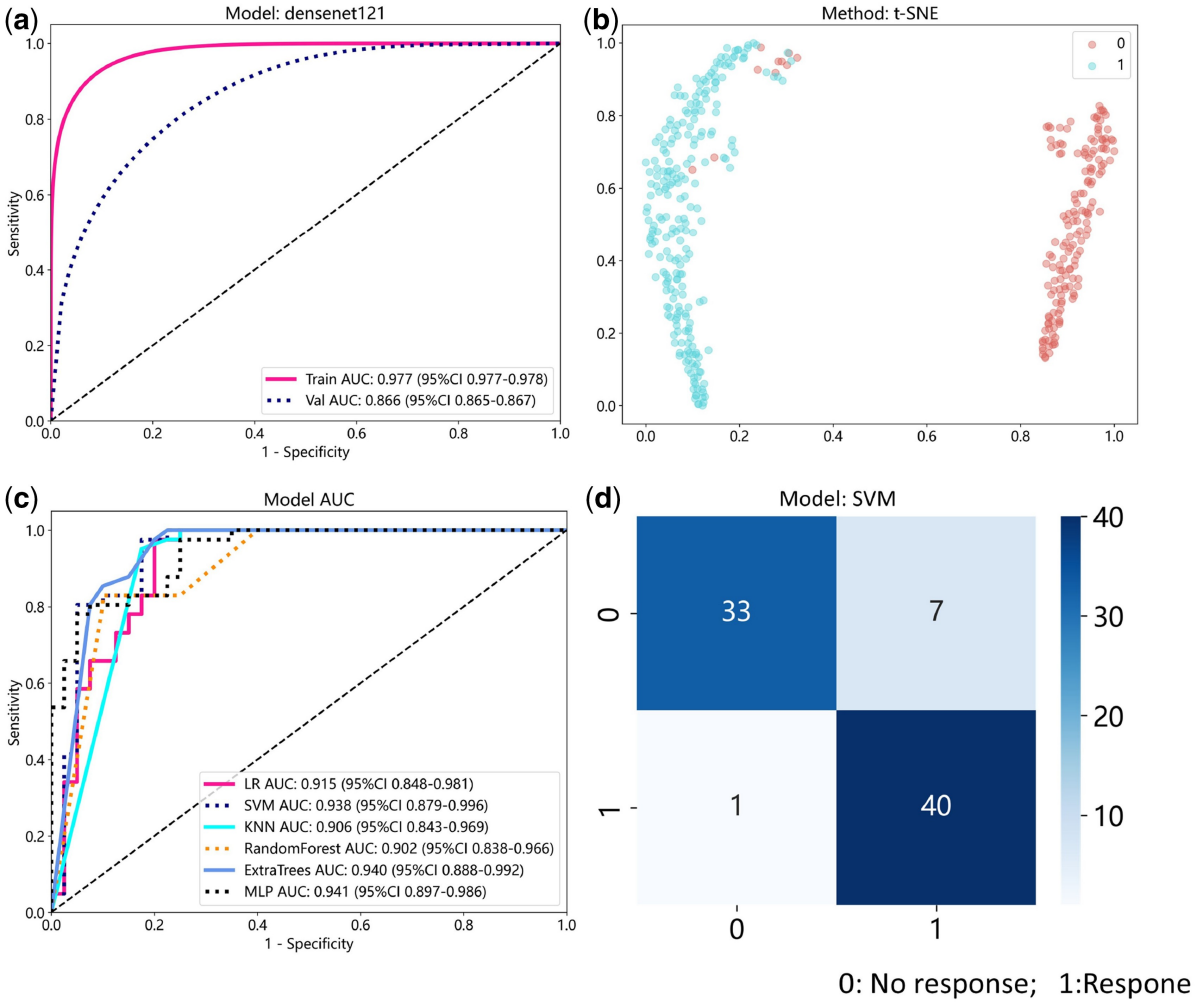
Evaluation of the patch-level model and the WSI-level model. (a) Prediction of UST responses at the patch level using the Densenet121 model. (b) t-SNE visualization of the distributions of UST response and non-response cases in two-dimensional space. (c) AUC values from predictions made by all machine learning classifiers at the WSI level. (d) Confusion matrix of the model’s test results in the test set.

The primary goal of screening patches is to select the most effective ones related to drug response. Figure 1b-2 shows the main algorithm flow. Our developed K-means (Wang *et al.* 2023) clustering algorithm model divides all patches of WSIs in the model cohort into K clusters. Next, K-independent patch-level CNN classifiers are trained on the K patch clusters from all patients in the model cohort, using Resnet50 as the CNN architecture, and the training parameters are the same as the all-patches classifier. RGB channels are normalized using z-score normalization while training each patch-level CNN classifier to ensure standardized image intensities suitable for model inputs. Data augmentation techniques, such as horizontal and vertical random flipping, are also employed during model training. Each cluster classifier exhibits varying abilities in classifying drug responsiveness. We use all patch classifiers as a performance benchmark. Clusters achieving higher classification accuracy than the benchmark are selected for further analysis. After dimensionality reduction through PCA, these clusters can be well distinguished in a two-dimensional space. Figure 1b-3 and b-4 presents the visualization results.

### 2.5 Prediction model for response to UST therapy

#### 2.5.1 Patch-level response prediction model for UST treatment

This study contains two levels of prediction: patch level and WSI level. Considering the significant size and variability of WSIs, we first segment the WSIs into patches. Subsequently, a MIL algorithm is employed to aggregate patch likelihoods to obtain WSI-level predictions. Following patch selection, some effective cluster patches from all samples were chosen to construct a UST response model for CD. WSI-level labels are utilized to establish a patch-level response model of UST response for CD. Figure 1c shows the pipeline for training the deep learning model for predicting patch-level UST response. Various deep-learning neural networks, including Mobilenet_v2, Resnet18, Resnet50, Densenet121, and Vision Transformer (ViT), were evaluated for patch-level prediction. We aim to determine the probability that each patch corresponds to its respective WSI label. Except for the ViT model, the prediction performance of other models at the patch level is similar. Therefore, this study adopts the Densenet121 model for all experiments.

Transfer learning was implemented to enhance model generalization across a heterogeneous cohort. This requires initializing the model using pre-trained weights from the ImageNet dataset while preserving the weights of the patch-level discriminator. Afterwards, the entire model was fine-tuned using a limited dataset (321 biopsy samples in the model set), which required weak annotations for our task. Transfer learning leveraged insights from ImageNet effectively and tailored them to fit the requirements of our classification problem. Densenet121 was employed in this study to build a patch-level drug response model. Generalization was enhanced by carefully adjusting the learning rate using the cosine decay learning rate algorithm. The learning rate is expressed as:


(1) $$n_{t}^{\mathrm{task}}=n_{\min}^{i}+\frac{1}{2}(n_{\max}^{i}-n_{\min}^{i})(1+\text{cos}(\frac{T_{\mathrm{cur}}}{T_{i}}\;\;\pi ))$$

$n_{\min}^{i}=0$ , $n_{\max}^{i}=0.01$, $T_{i}=30$ represent the minimum learning rate, the maximum learning rate, and the number of iteration epochs, respectively. Our vast data set contains more than two million training patches, enabling the model to achieve better results in neural networks of different structures. Additionally, we utilize transfer learning algorithms to ensure optimal model fit. The parameters of the backbone are initialized by loading the pre-trained model, allowing the parameters of the current task to be effectively fine-tuned. When the number of training reaches $1/2T_{i}$, the cosine decay learning rate algorithm is performed on the Backbone’s learning rate. The learning rate of the backbone component is defined as:


(2) $$n_{t}^{\text{backbone}\;}=\{\begin{array}{cc} 0 & \;\text{if}\;T_{\text{cur}\;}\leq \frac{1}{2}T_{i}\\ n_{t}^{\mathrm{task}} & \;\text{if}\;T_{\text{cur}\;}>\frac{1}{2}T_{i} \end{array}$$

Other hyperparameter configurations are as follows. The optimizer uses SGD, the loss function uses softmax cross-entropy, and the batch size is 256 pixels. We applied class weighting in the loss function based on inverse class frequencies to address class imbalance during patch-level model training.

#### 2.5.2 Multi-instance learning for WSI fusion

The patch-level model predicts labels and probabilities for all patches. Subsequently, MIL was employed to combine these patch probabilities, training a classifier model to generate predictions at the WSI-level. Patch likelihoods were derived using two distinct machine-learning methods. (i) Patch Likelihood Histogram (PLH) Pipeline: This approach employs histograms to represent the distribution of patch likelihoods within WSIs. Discretizing the likelihoods effectively captures the distribution of likelihoods as a representation of WSI. (ii) Bag of Words (BoW) pipeline: The BoW pipeline is built on histogram-based and vocabulary-based techniques. It uses term frequency-inverse document frequency (TF-IDF) mapping for each patch, resulting in TF-IDF feature vectors representing WSI. These feature vectors are then used to train traditional machine learning classifiers to predict each WSI’s label. Utilizing two independent pipelines effectively consolidated the initially disparate patch-level predictions, generating WSI-level features that provide valuable information for subsequent analytical procedures. Figure 1d-1 shows the pipeline for WSI feature aggregation.

#### 2.5.3 Signature building

The feature splicing is constructed based on the PLH and BoW pipelines. Due to the complex feature distribution, to control the model’s complexity, improve the model’s generalization ability, and enhance the stability, this study employs the *z*-score regularization method on the features. This regularization transforms the data into a mean of 0 and a variance of 1. The regularization formula is expressed as:


(3) $$n_{\mathrm{col},\mathrm{row}}=\frac{a_{\mathrm{col},\mathrm{row}}-\mathrm{mea}n_{\mathrm{row}}}{std_{\mathrm{row}}}$$

$n_{\mathrm{col},\mathrm{row}}$ is the regularized value, $a_{\mathrm{col},\mathrm{row}}$ is the original value, $\mathrm{mea}n_{\mathrm{row}}$ is the mean of a particular dimension feature, and $std_{\mathrm{row}}$ is the standard deviation of a particular dimension feature.

To handle high-dimensional features, we first apply the Pearson correlation coefficient to select features strongly associated with the prediction target greater than 0.9, followed by LASSO with cross-validation using MSE for refinement. The final feature set trains various ML models, including LR, SVM, KNN, RF, ExtraTrees, and MLP, for UST response prediction in CD. The process is illustrated in Fig. 1d-2 and e, with model comparisons shown in Fig. 1e. Our pipeline, while effective, involves multi-stage processing steps that incur substantial computational overhead. For example, preprocessing and patch extraction took around 48 h using an NVIDIA GeForce RTX4090 GPU, and patch-level model training selected clusters required an additional 16–20 h. Although these steps can be parallelized, such resource requirements may hinder large-scale deployment in real-world clinical settings. Future work will explore more streamlined or end-to-end variants to reduce complexity and improve efficiency.

## 3 Results

### 3.1 Cluster analysis of patches

First, the original image is preprocessed to convert WSI into patches of 256×256 pixel size. The acquired patches are then normalized. This article uses the Vahadane colour normalization method. The differences with the other two colour normalization methods are visualized in Supplementary Fig. S2 (Reinhard *et al.* 2001, Macenko *et al.* 2009). It should be noted that the Vahadane (Vahadane *et al.* 2016) colour normalization method is relatively inefficient, so it takes a long time to perform colour normalization on millions of patches.

We randomly selected 50 samples from our dataset. 329 045 patches were generated as the dataset for the k-means clustering algorithm used in this study. The CH index was used to evaluate the optimal number of clusters. The CH index is calculated as the ratio of between-cluster variance to within-cluster variance. A higher CH index value signifies better clustering performance, indicating well-separated and distinct clusters. Among the three clusters, Cluster 3 yielded the highest classification accuracy. Cluster 3 predominantly contained tissue regions with concentrated inflammatory infiltrates and preserved glandular structures, features which are more strongly associated with UST treatment response. As shown in Fig. 3a and b, the CH index reached its highest when the clusters were divided into three categories in the study. The CH index reached its highest value of 3039.4.

### 3.2 Patch selection-based deep learning framework

All patches are divided using the optimal number of clusters determined by the k-means algorithm, and the dataset is divided into three clusters. Then, the CNN model is employed to build a patch-level classifier, yielding three independent classifiers corresponding to each cluster. The baseline accuracy achieved by this study’s CNN model reaches 0.72. Specifically, the accuracy rates for cluster 1, cluster 2, and cluster 3 are 0.68, 0.76, and 0.79, respectively. Consequently, clusters 2 and 3 are selected to build the response model due to their higher accuracy in predicting treatment response, as illustrated in Fig. 3c.

We optimize patch selection through a clustering strategy to build a classification model based on UST response. About 2 million patches were selected in the training set, among which the patches extracted from clusters 2 and 3 significantly outperformed the baseline classifier in the responsiveness classification task (indicated by the green columns in Fig. 3c). Compared with cluster 1, which contains edge artefacts and background or low-information areas, clusters 2 and 3 are richer in glandular structures and inflammatory cell areas related to treatment response and have higher discriminability. This strategy not only improves the accuracy and interpretability of the model but also effectively reduces the computational overhead and noise interference during training. Figure 3d displays the clustering results for three representative WSIs. Additional details and visualizations of multiple consecutive sections for these patients are provided in Supplementary Fig. S1, emphasizing the consistent clustering results and the efficacy of clustering-based methods in distinguishing diverse image patterns.

### 3.3 Evaluation of patch-level model performance

We compare the models using patch-level AUROC to evaluate the accuracy of the pathological model in regional recognition. The model was assessed by Mobilenet_v2, Resnet18, Resnet50, Densenet121, and Vision Transformer (ViT). The performance of these deep learning models is detailed in Supplementary Table S1. Each model’s performance is evaluated based on ROC curves and AUC values. Except for the ViT model, the performance of the other models was similar. In the test cohort, the Densenet121 model used in this study had a patch-level AUROC of 0.866 (95% CI: 0.865–0.867) for diagnosing whether CD responded to UST. Figure 4a illustrates the AUROC of the Densenet121 model. The AUROC of other models are shown in Supplementary Fig. S3.

**Figure 5.**
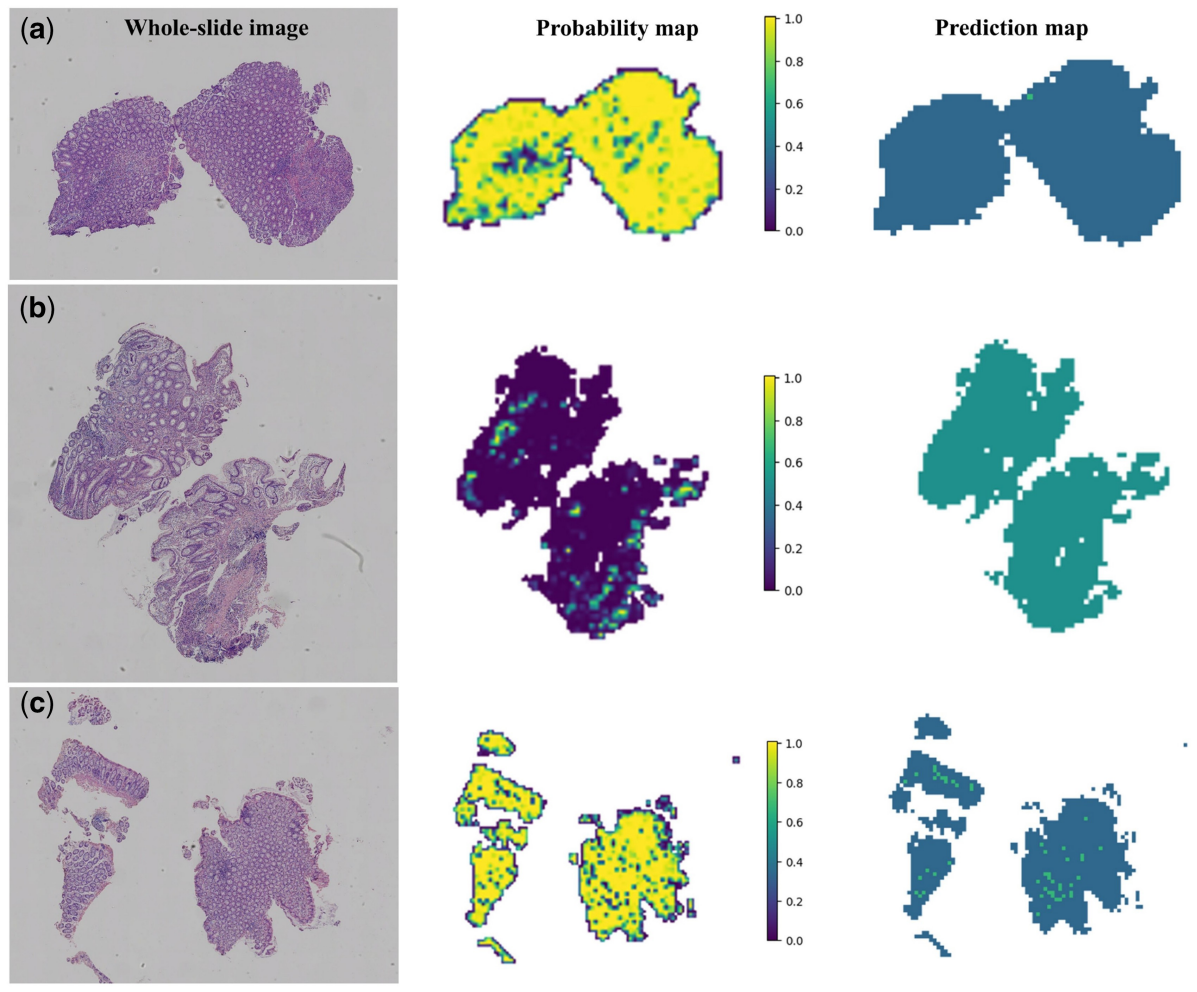
Prediction probability map and prediction heat map of the UST response model for CD patient treatment at the patch level. The figure shows the H&E slide at the WSI level (left), the heatmap of the predicted probability for each patch (probably map with probability label, middle), and the resulting prediction map of the WSI (right). Panel (a) shows a WSI responding to UST. Panel (b) depicts a WSI that does not respond to UST. (c) Illustrates a WSI that responds to UST, yet contains many non-responsive patches in the prediction image.

To further visualize and explain the performance of the deep learning model at the patch-level of UST response in CD patients, Fig. 5 displays predicted labels and probability heatmaps for evaluation. The visualization results demonstrate the high accuracy of the pathological prediction model in this study when evaluating patches. In addition, to further explain the prediction results of the model, Grad-CAM is used to visually display the gradient of the last convolutional layer of the network, generating a heatmap that highlights class-specific spatial information. Notably, it does not require any modifications to the model architecture or additional training. Supplementary Fig. S4 illustrates the use of Grad-CAM for visualizing activations of the last convolutional layer in diagnostic class evaluation. This transparent description highlights regions of the input image that contribute significantly to predictions, providing valuable insights into the model’s decision-making process.

### 3.4 Evaluation of WSI-level model performance

#### 3.4.1 t-SNE visualization of WSI distributions

During the development of the predictive model, we derived a feature set by including the predicted labels individually as histogram features. To understand how patch-level features aggregate into WSI-level representation, we adopt the t-SNE algorithm (Fig. 4b). Interestingly, clear differentiation was observed between the responder and non-responder groups when plotted in two-dimensional space. In addition, this study aggregates patches into two WSI-level feature groups (BoW and PLH). This study uses the t-SNE algorithm to draw it in two-dimensional space (Supplementary Fig. S5), and apparent differentiation can be observed for both sets of features. Therefore, the two sets of features obtained by aggregating patches using BOW and PLH effectively predict patient UST treatment responses.

#### 3.4.2 Selection of discriminative features

The feature splicing built based on the two pipelines of PLH and BoW comprises 202 dimensions. Higher dimensions are not conducive to the training of models. Therefore, this study initially uses the *z*-score method to standardize the feature set. Subsequently, the Pearson correlation coefficient is calculated for each dimension feature to identify correlations. Features with a correlation coefficient greater than 0.9 are filtered out. A total of 45-dimensional features were obtained, and the correlation between different features was displayed by visualizing the cluster analysis matrix of feature correlation coefficients (Supplementary Fig. S6a).

In addition, the selected 45-dimensional features are used for feature selection again using the LASSO model. Use the cross-validation method to select the weight of features under different lambdas (Supplementary Fig. S7a). The performance of the LASSO model was concurrently evaluated (Supplementary Fig. S7b). A histogram showing the weights of the filtered features is shown in Supplementary Fig. S6b. Features with zero weights were removed.

#### 3.4.3 Performance evaluation of the predictive model

Based on the selected features, the 5-fold cross-validation method was used in the model set to verify and train six different machine learning models: LR, SVM, KNN, RF, ExtraTrees, and MLP. The performance metrics of the five-fold cross-validation of all models are shown in Supplementary Table S2, and their ROC curves and AUC values are shown in Fig. 4c. LR, SVM, and MLP exhibited better performance, with all evaluation metrics in the model set reaching high levels, achieving an accuracy of 0.997. In the test set, SVM, LR, and MLP accuracies were 0.889, 0.877, and 0.852, respectively. Conversely, KNN, RF and ExtraTrees models showed poor performance for the current task, low sensitivity, and inability to predict responsiveness. According to test set performance evaluation indicators, the SVM model performs best with an accuracy of 0.889 and an AUC of 0.938 (95% CI: 0.879–0.996). It achieved a sensitivity of 0.951, a specificity of 0.825, and an F1 score of 0.897. The LR model showed performance close to SVM, albeit with slightly lower specificity. The MLP model had lower sensitivity and accuracy compared to SVM.

Decision Curve Analysis (DCA) is an essential indicator for evaluating model performance. We further evaluate the model using DCA to predict whether a sample is responsive in the test set. As shown in Supplementary Fig. S7c, DCA indicated that the models performed well and possessed substantial clinical application value. The probability histogram of whether CD responds to UST for all test set samples diagnosed by the SVM model is shown in Supplementary Fig. S7d. Among them, seven non-responsive samples were predicted to be responsive, and one responsive sample was predicted to be non-responsive. Figure 4d visually displays the test sample prediction results using a confusion matrix. In addition, DCA was used to evaluate the performance of the LR and MLP models in the test set. The sample prediction probability histogram and confusion matrix are shown in Supplementary Fig. S8.

## 4 Discussion

Computational pathology (CPath) employs deep learning methods and algorithms to analyse pathological images. Due to the development of AI technology, many is crucial applications in CPath have emerged (Verghese *et al.* 2023), ranging from automating diagnostic and screening tasks to mining tissue microenvironmental signatures for prognostic analysis, treatment response prediction, and predictive biomarkers (Boehm *et al.* 2022, Yang *et al.* 2022, Zhang *et al.* 2022, Foersch *et al.* 2023, Bashir *et al.* 2023). While pathologists diagnose diseases by examining tissue samples through biotechnology, diagnostic decisions heavily rely on pathologists’ extensive experience. However, challenges such as the increasing demand for skilled pathologists and rising workloads necessitate the development of tools to support routine diagnostic tasks and promote early screening. AI technology plays a crucial role in advancing pathology towards precision medicine (Verghese *et al.* 2023).

In prior studies, many research efforts employing AI techniques to forecast drug treatment responses focused on cancer. Fewer studies have delved into predicting drug treatment responses in non-cancerous diseases, marking this research pioneering in predicting UST treatment responses for CD. This research introduces an AI-driven method for forecasting the UST treatment response model, enabling the automated prediction of UST efficacy response in CD patients using unannotated WSI. The study analysed 404 endoscopic biopsy intestinal tissues. Extensive validation and comparative analyses were conducted, providing robust evidence for the accuracy and generalizability of our model.

The model we developed is dedicated to learning the most representative features from WSIs, offering two main advantages. First, it automatically clusters similar samples through a clustering algorithm. Then it selects several type-related image clusters that contribute more to the classification task, avoiding the need for any manual annotation. Second, it selectively fuses the most discriminative information in relevant patches and performs multi-instance aggregating local features to obtain a global diagnosis. In traditional supervised deep learning training, regions of interest need to be manually marked. However, manual annotation is time-consuming and subjective. To avoid pixel-level annotations, weakly supervised methods were developed where experts can assign labels to WSIs. Among them, MIL and its variants using the ‘bag learning’ strategy have been widely used in WSI classification tasks, such as ABMIL (Ilse *et al.* 2018), PMIL (Yu *et al.* 2023), and CLAM (Lu *et al.* 2021) and other algorithms. These algorithms are based on WSI prediction and do not discriminate patches. Due to the high variability and complexity of pathology data, there is much noise in the data, making it difficult to discover effective attention weights for instances related to the target category, thus affecting the model’s performance. Supplementary Table S3 shows the results of using the current state-of-the-art MIL methods on the task in this paper. This study selects effective image clusters for multi-instance fusion by identifying the relationship between each image cluster and UST therapeutic response. The study finds that superficial and small tissue areas are excluded, while glands and tissue areas where cells accumulate are retained. These have an essential impact on predicting the therapeutic response of UST.

Based on the training of different types of deep learning models based on transfer learning and massive data sets, it was found that different deep learning models achieved relatively high accuracy in predicting UST therapeutic response at the patch level. Therefore, this study selects the Densenet121 model with the highest accuracy in the independent test set to predict UST therapeutic response at the patch level. The UST therapeutic response prediction results at the patch level are mapped to the UST therapeutic response prediction at the WSI level. This study employs PLH and BoW features for multi-instance fusion prediction and conducts separate analyses to predict the therapeutic response of UST for these two sets of features. Both sets of features play a particular role in predicting the therapeutic response of UST.

We collect biopsy pathological images of patients pre-treated with UST and use a classifier to predict whether patients treated with UST will respond. Five-fold cross-validation is used to evaluate the classifier’s performance in the model set. It is impossible to distinguish which machine learning classifier performs better from the AUC value. Afterwards, by calculating the accuracy, sensitivity, specificity, positive predictive value (PPV) and negative predictive value (NPV) of the model, it was found that LR, SVM and MLP were more suitable for the prediction of treatment response in this study (Supplementary Table S2). LR, SVM and MLP are traditional supervised learning classifiers suitable for different problems. KNN, RF and ExtraTrees are classifiers in ensemble learning that improve classification performance by combining multiple base classifiers. This study is a two-classification problem, so ensemble learning is not suitable for the research questions of this study. Five-fold cross-validation was utilized to evaluate the performance indicators of LR, SVM and MLP, and the comparison shows that the performance of the SVM classifier is slightly better than the other two classifiers (Supplementary Table S2). Ablation studies across six configurations show that PLH and BoW features improve WSI prediction, with their fusion performing best in the Supplementary Table S4. Pearson correlation coefficient filtering and LASSO enhance generalization. Our pipeline achieved the highest AUC and F1 scores. Therefore, the constructed model has a high accuracy in predicting whether patients treated with UST will respond, which will help doctors achieve personalized and precise treatment for CD patients. Furthermore, the graphical representation of the DCA clearly shows that the constructed model yields a net benefit on this study’s dataset. The confusion matrix results showed that the model had high sensitivity and could accurately predict patients with CD who would respond to UST. The probability histogram shows the only sample in the test set where the UST response is predicted to be false negative. The sample is predicted to be non-responsive to UST. The probability histogram shows that the probability of non-response is small. However, this study only investigated a single-centre sample, and a multicentre cohort should be considered in the future to validate the model’s performance.

In conclusion, we developed a model for predicting UST efficacy response in CD patients through digital pathology-based AI, which showed competitive performance in the test cohort. Therefore, our model can be considered for predicting UST efficacy response in patients with CD, and the model’s prediction results can guide doctors’ treatment in the future.

## Supplementary Material

btaf301_Supplementary_Data

## Data Availability

Restrictions apply to the availability of the in-house data, which were used with institutional permission for the current study and are thus not publicly available. We note that these data were not specifically collected for this study. All requests for data may be addressed to the corresponding author and will be promptly evaluated based on institutional and departmental policies to determine whether the data requested are subject to intellectual property or patient privacy obligations. Internal data can only be shared for noncommercial, academic purposes and will require a data user agreement.
