# Supplementary material for: Predicting ustekinumab treatment response in Crohn’s disease using pre-treatment biopsy images
Source: Bioinformatics. 2025 May 14;41(6):btaf301. doi: 10.1093/bioinformatics/btaf301 (PMC12133262; doi:10.1093/bioinformatics/btaf301)
Supplement: btaf301_Supplementary_Data [file btaf301_supplementary_data.zip › Supplementary material.pdf]

Supplementary material

Predict Ustekinumab Response Based on Pre-Treatment Biopsy Pathology Images from Crohn's Disease Patients

Chengfei Cai[1,2,3]*, Ruidong Chen[4]*, Jieyu Chen[5]*, Jun Li[2], Caiyun Lv[4], Yiping Jiao[2], Lanqing Wu[6], Juan Chen[6], Han Xu[4], Qi Sun[5], Qianyun Shi[5], Jun Xu[2#], Wen Tang[4#], Yao Liu[4,5,6#]

[1] School of Automation, Nanjing University of Information Science and Technology, Nanjing 21004, China
[2] Institute for AI in Medicine, School of Artificial Intelligence, Nanjing University of Information Science and Technology, Nanjing 21004, China
[3] College of Information Engineering, Taizhou University, Taizhou 225300, China
[4] Department of Gastroenterology, the Second Affiliated Hospital of Soochow University, Suzhou 215004, China
[5] Department of Pathology, Nanjing Drum Tower Hospital, the Affiliated Hospital of Nanjing University Medical School, Nanjing 210008, China
[6] Department of Pathology and Pathophysiology, Medical College of Soochow University, Soochow University, Suzhou 215123, China

*Chengfei Cai, Ruidong Chen and Jieyu Chen contributed equally to the work.

#Correspondence:
Jun Xu, Ph.D., Professor, Institute for AI in Medicine, School of Artificial Intelligence, Nanjing University of Information Science and Technology, Nanjing 21004, China; E-mail: jxu@nuist.edu.cn

Wen Tang, M.D., Doctor, Department of Gastroenterology, the Second Affiliated Hospital of Soochow University, Suzhou 215004, China; E-mail: louisetangwen@163.com

Yao Liu, M.D., Ph.D., Doctor, Associate Professor, Department of Pathology, Nanjing Drum Tower Hospital, the Affiliated Hospital of Nanjing University Medical School, Nanjing 210008, Jiangsu, China; E-mail: yao.liu@njglyy.com or yliu1206@suda.edu.cn

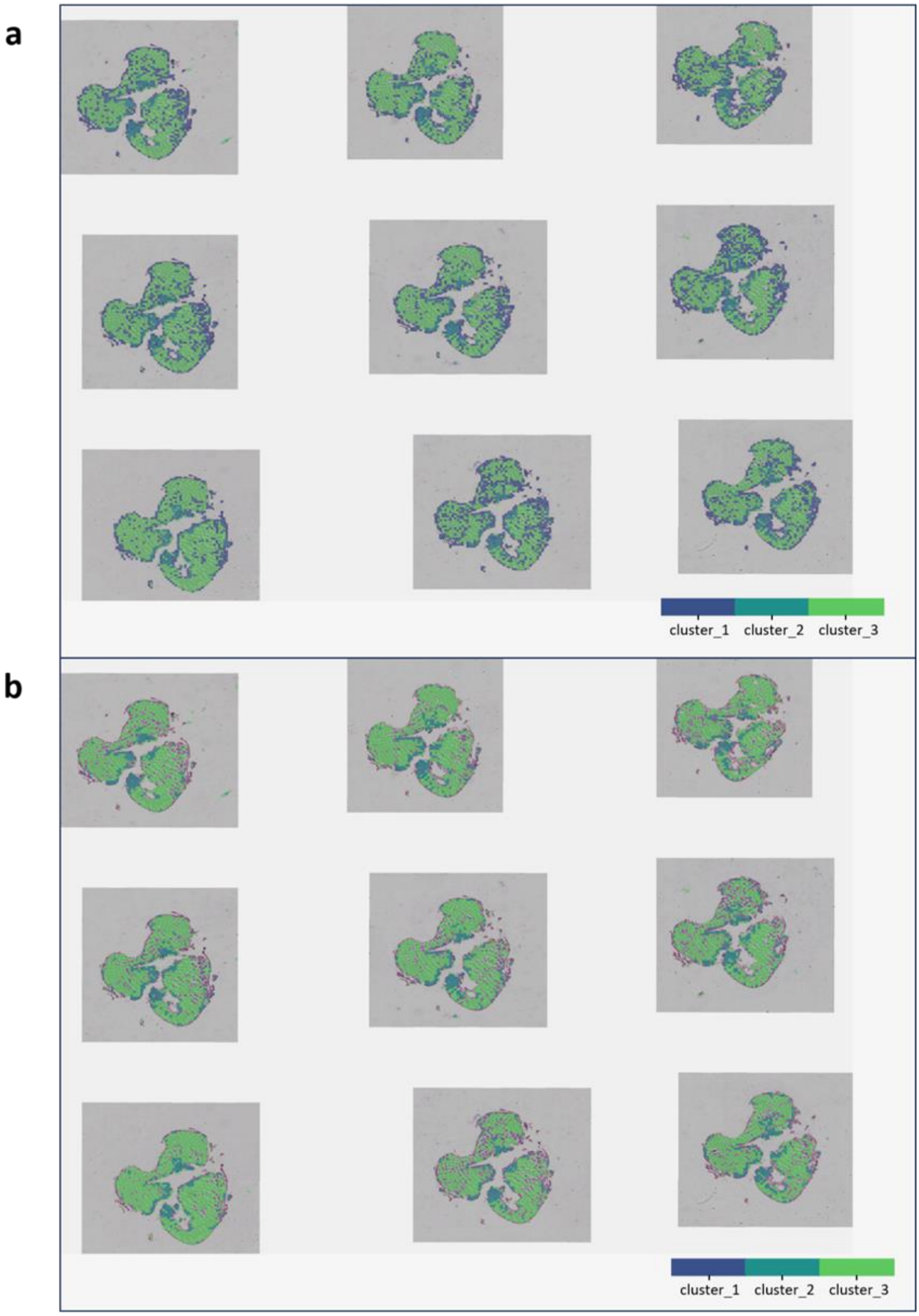

Supplementary Figure 1. Results of patch clustering and selection for serial sections of a single tissue sample: (a) the result of image patch clustering and (b) the result of image patch selection.

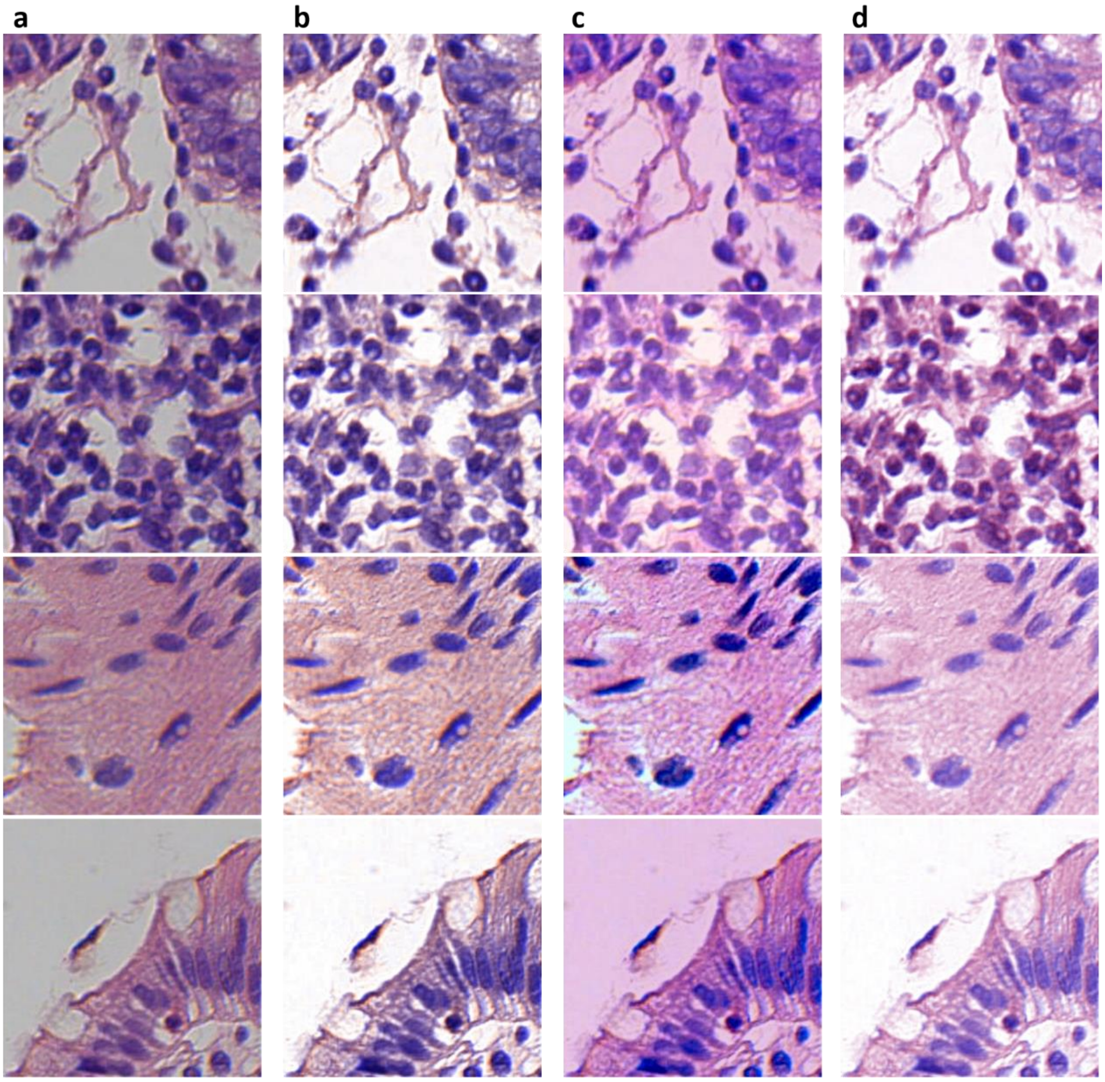

Supplementary Figure 2. Visualization of the effect of the color normalization method. (a) Original image patch; (b) Normalized result of Macenko method; (c) Normalized result of Reinhard method; (d) Normalized result of Vahadane method.

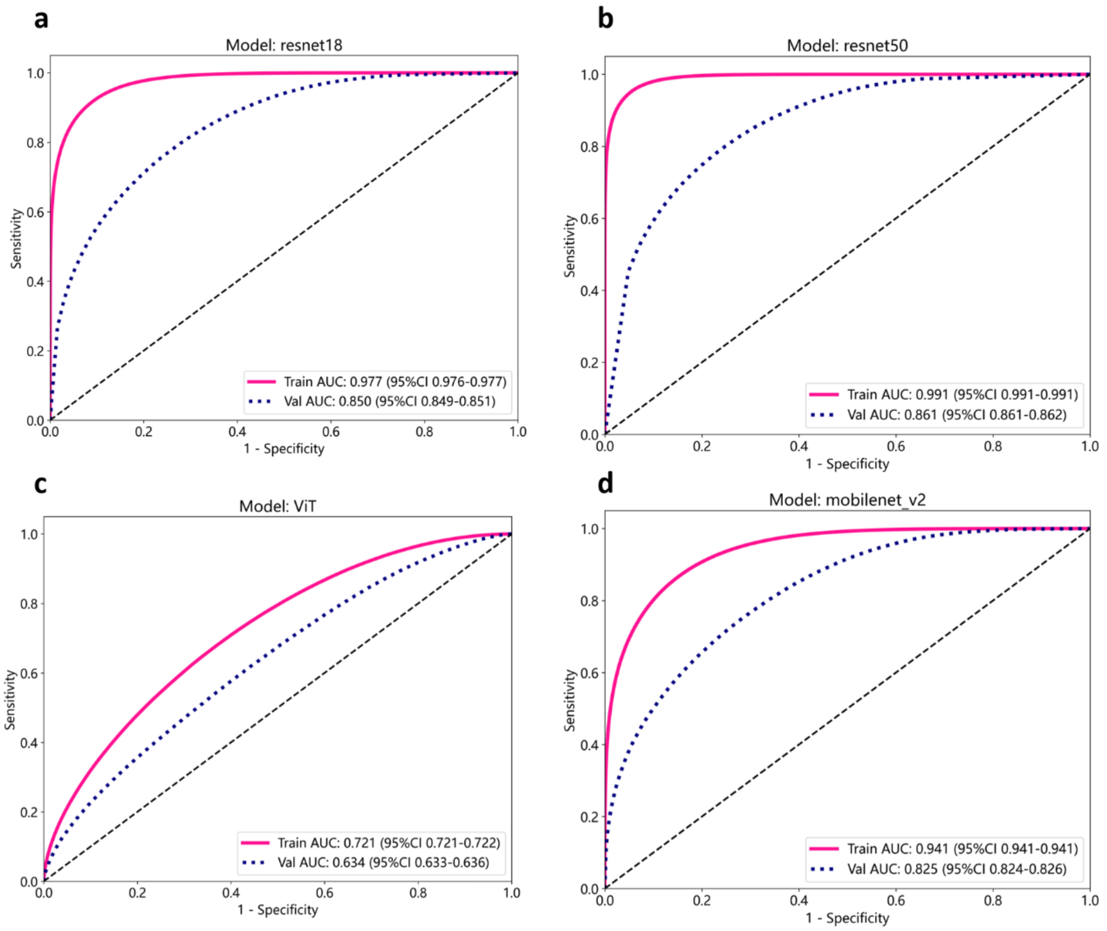

Supplementary Figure 3. Evaluation of the results of the deep learning model predicting UST response to CD at the image patch level. (a), (b), (c), and (d) are the performance evaluation results of Resnet18, Resnet50, ViT, and mobilenet_v2 models, respectively.

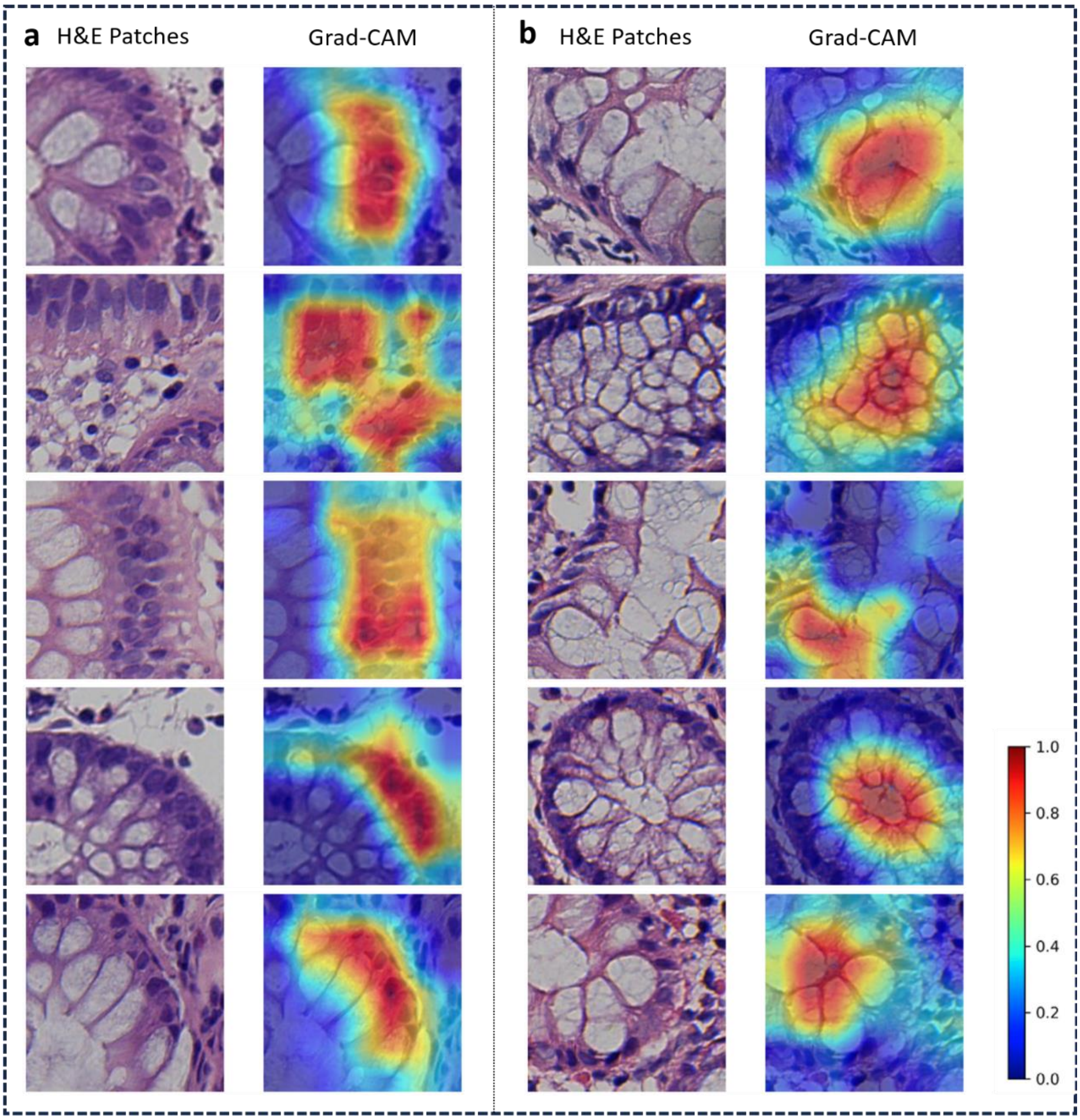

Supplementary Figure 4. Visualizes the last layer convolution results of the predicted deep learning model using the Grad-CAM method. (a) is the result of the patch with a response; (b) is the result of the model prediction without a response

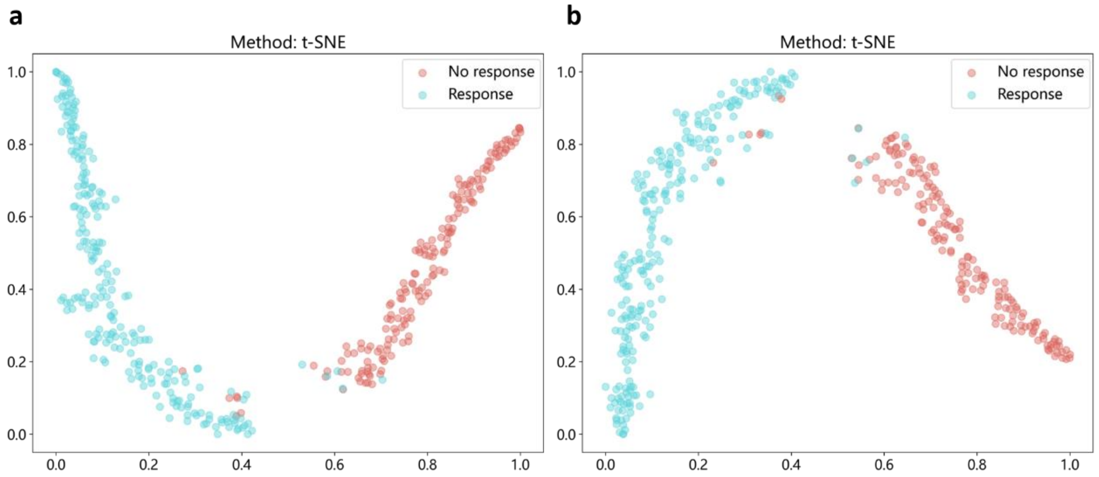

Supplementary Figure 5. t-SNE algorithm for plotting UST response and non-response distributions in two-dimensional space for the two sets of features, PLH and Bow. (a) is the visualization result of PLH; (b) is the visualization result of BoW.

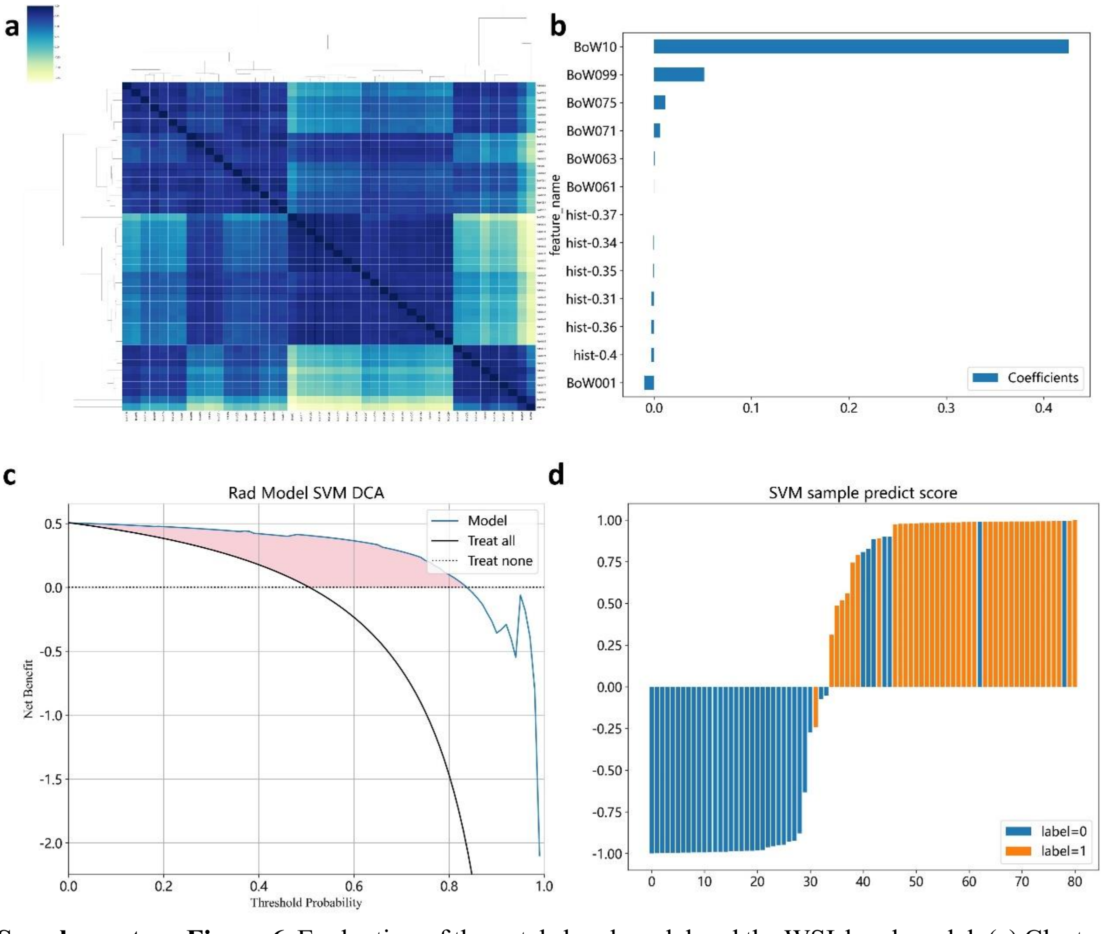

Supplementary Figure 6. Evaluation of the patch-level model and the WSI-level model. (a) Cluster analysis of Pearson correlation coefficient of pathological features based on deep learning at the patch level. (b) Features weights determined by the LASSO algorithm. (c) DCA of the SVM classifier. (d) Predicted probability values for each tissue sample in the test set.

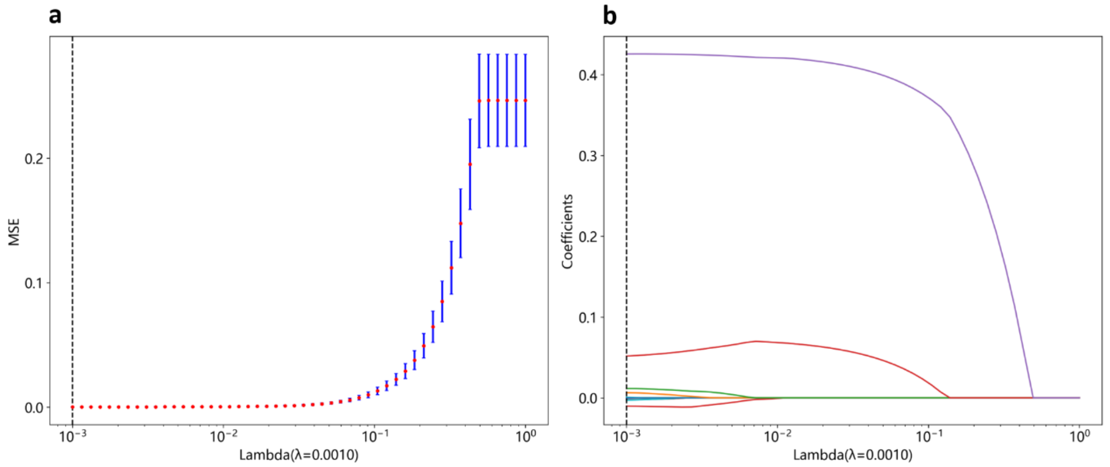

Supplementary Figure 7. The LASSO regression model selects strongly correlated features. (a) Select the optimal λ value based on 5-fold cross-validation and minimum mean square error (MSE), represented by the vertical dashed line. (b) Shows the LASSO coefficients for different λ values, where the vertical dashed line indicates the number of features corresponding to the optimal λ value.

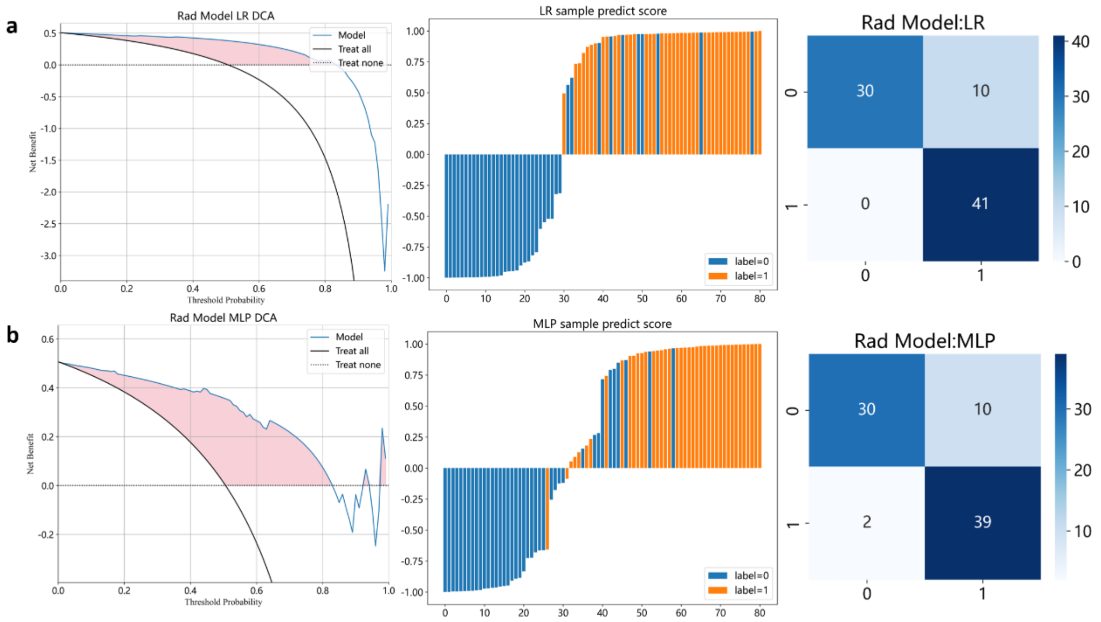

Supplementary Figure 8. Performance evaluation of LR and MLP models. (a), (b) respectively show the performance of LR and MLP models in predicting the response of UST to CD patients, including the DCA curve, the prediction probability histogram of each sample in the test set, and the confusion matrix of the model prediction for all test samples.

Supplementary Table 1. Results of evaluating the performance of five deep learning models at the patch level in predicting the response of UST.

Model	Cohort	ACC	AUC	95% CI	Sensitivity	Specificity	PPV	NPV	F1
Densenet121	Train	0.916	0.977	0.9773-0.9776	0.920	0.910	0.942	0.879	0.931
	Test	0.775	0.866	0.8653-0.8670	0.807	0.746	0.743	0.809	0.774
Mobilenet_v2	Train	0.860	0.941	0.9406-0.9412	0.868	0.847	0.899	0.803	0.883
	Test	0.733	0.825	0.8244-0.8264	0.749	0.718	0.708	0.758	0.728
Resnet18	Train	0.914	0.977	0.9764-0.9767	0.916	0.910	0.941	0.874	0.929
	Test	0.759	0.850	0.8488-0.8507	0.763	0.755	0.740	0.777	0.752
Resnet50	Train	0.950	0.991	0.9913-0.9914	0.953	0.944	0.964	0.928	0.959
	Test	0.775	0.861	0.8605-0.8623	0.797	0.755	0.748	0.803	0.772
ViT	Train	0.660	0.721	0.7207-0.7219	0.678	0.743	0.743	0.555	0.709
	Test	0.587	0.634	0.6331-0.6358	0.619	0.561	0.561	0.616	0.588

Supplementary Table 2. Results of evaluating the performance of 6 machine learning classifier models in predicting response to UST efficacy in CD patients in the test set.

Model	ACC	AUC	95% CI	Sensitivity	Specificity	PPV	NPV	F1
LR	0.877	0.915	0.8481 - 0.9812	0.951	0.800	0.830	0.941	0.886
SVM	0.889	0.938	0.8794 - 0.9962	0.951	0.825	0.848	0.943	0.897
KNN	0.494	0.906	0.8429 - 0.9687	0.000	1.000	0.000	0.494	-
RF	0.852	0.902	0.8376 - 0.9661	0.805	0.900	0.892	0.818	0.846
ExtraTrees	0.864	0.940	0.8882 - 0.9922	0.878	0.850	0.857	0.872	0.867
MLP	0.852	0.941	0.8965 - 0.9864	0.756	0.950	0.939	0.792	0.838

Supplementary Table 3. Experimental results of the end-to-end MIL models in predicting UST treatment response

Method	Accuracy	AUC	Precision	Recall
ABMIL	70.13±4.66	68.85±4.43	76.36±3.64	72.78±7.01
CLAM	71.40±4.34	68.63±4.14	74.34±3.50	79.19±6.03
TransMIL	67.17±2.35	62.38±2.46	70.10±3.22	78.02±8.96
DSMIL	66.67±3.12	68.40±3.87	76.37±3.64	68.78±3.98
DTFD-MIL	68.65±2.57	70.01±4.24	75.20±3.54	70.37±8.14
R^2T-MIL	71.89±5.05	72.77±2.90	77.97±2.96	73.24±3.62

Supplementary Table 4. Ablation experiment comparing the use of PLH and BoW features alone and in combination with feature selection

Feature	ACC	AUC	95% CI	Sensitivity	Specificity	PPV	NPV	F1
PLH	0.877	0.905	0.8425 - 0.9812	0.922	0.850	0.860	0.895	0.881
PLH (no selection)	0.864	0.879	0.8068- 0.9517	0.927	0.800	0.926	0.914	0.874
BoW	0.879	0.907	0.8338 - 0.9796	0.926	0.820	0.833	0.913	0.879
BoW (no selection)	0.864	0.880	0.8087- 0.9523	0.927	0.800	0.926	0.914	0.874
PLH+BoW (no selection)	0.877	0.880	0.8077 - 0.9520	0.925	0.815	0.830	0.921	0.886
PLH+BoW+feature seclection	0.889	0.938	0.8794 - 0.9962	0.951	0.825	0.848	0.943	0.897
